# Diversity in tumor territory of meningioma: Protein expression in vascular endothelial growth factor and epidermal growth factor

**Published:** 2019-04-04

**Authors:** Parvin Mehdipour, Firoozeh Javan, Morteza Faghih-Jouibari, Mehdi Khaleghi

**Affiliations:** 1 Department of Medical Genetics, School of Medicine, Tehran University of Medical Sciences, Tehran, Iran; 2 Shariati Hospital, School of Medicine, Tehran University of Medical Sciences, Tehran, Iran

**Keywords:** Vascular Endothelial Growth Factor, Epidermal Growth Factor, Meningioma, Protein Expression, Genetics Polymorphism, Molecular Evolution

## Abstract

**Background:** Vascular endothelial growth factor (VEGF) and epidermal growth factor (EGF) are involved in tumor development and progression. But, the classified-based data of protein expression (PE) in meningiomas is unavailable. Therefore, we aimed to explore the PE of VEGF and EGF in meningiomas by considering evolutionary strategy and the regional tumor-based assay.

**Methods:** PE was assayed using immunofluorescence (IF) within the peripheral, central, and basal sections of four meningioma tumors, and a lung metastatic brain tumor as a positive control.

**Results:** Diverse characteristics and harmonic cross-talk in the individual sections and between different tumor sections were traced. The mode of PE was puzzling and personalized issue. Co-expression had a key impact on tumor evolution and diverse PE profiles led to draw the heterogenic classification, as the personalized/complementary insight in the functional behavior of VEGF and EGF. D1853N polymorphism of ATM gene was mosaics in two patients with meningiomas.

**Conclusion:** The classified heterogeneity, harmonic co-expression, and diverse functional information in different regions of tumors may lead to predict the aggressiveness mode of tumors as a translational insight to the clinical managements including therapy in brain tumors.

## Introduction

The most challenging item in cancer is heterogeneity through which the tumor capability leads to the diverse characteristics in different neoplasms. So, the layer to layer, and cell to cell exploration may unmask the road map of cancer initiation and progression.

Meningiomas, as the intradural spinal tumors,^1^ are the most frequent primary neoplasms in adults,^2^ of those 80% are classified as benign tumors.^3^ Based on an update, brain invasion is considered as a typical meningioma, World Health Organization (WHO) grade II.^4^ It was reported that the frequency of meningioma amongst different types of brain tumors is revealed to be 36% in adults.^5^

Driving mutation and the successful production of truncated proteins is puzzling. So, by highlighting the importance of target-based therapy, protein expression (PE) assay would lead to an innovative approach to consider a reliable choice for cancer therapy.^6^ Besides, progression of meningioma relies on edema and growth factor. Strategy of vascular system is quite complicated.^7^ Furthermore, vascular endothelial growth factor (VEGF) family is capable to organize tumor vascularization and is, basically, originated through the evolution in non-vertebral species.^8^ Epidermal growth factor (EGF) has the key role in differentiation, cell proliferation, tumorigenesis, and apoptosis. Besides, the EGF receptors (EGFRs) are overexpressed in most of the epithelial tumors.^9^ Overexpression of EGF is found to be associated with development, progression, invasiveness, and metastasis which are indicative of poor prognosis in cancer.^10^ Besides, the mode of PE is found to be different in brain tumors comprising five meningiomas.^11^

D1853N polymorphism has been reported as a predisposing factor either in brain tumors by providing three-hit hypothesis,^12^ or in breast cancer.^13^ Recently, a novel ‘Five-hit hypothesis’ and the key role of the D1853N polymorphism in breast cancer evolution is published.^14^ Besides, according to the Single Nucleotide Polymorphism Database (dbSNP) and the Catalogue of Somatic Mutations in Cancer (COSMIC), the D1853N alteration was re-confirmed as a predisposing sequence variant. However, the aim of the present work was to explore the status of PE in VEGF and EGF in different regions of the meningioma tumor samples to trace diversity and evidences of an evolutionary event within the tumor’s regions.

## Materials and Methods

Designing of this investigation was based on an evolutionary and personalized insight. According to the en bloc removal, resection was performed in one piece; and all the patients were operated by the same method. Then, three samples were dissected from peripheral (Per), central (C), and basal (Ba) regions. The fresh tissues from four patients with meningioma and a lung metastatic brain carcinoma (LMBC), as a malignant/positive control, were investigated. The patients have not been endured by any therapy before surgery. Based on the strategy of the local ethics committee, all of the patients have signed the consent form. Four patients, pathologically diagnosed as the primary meningioma. The blood sample of a healthy control, without any family history of neoplastic disorders, was also included in this investigation. The characteristics of patients are presented (Table 1). 

**Table 1:** Characteristics of the patients

**Patients’ ID**		**Age patient ** **(year)**	**Gender**	**Histopathology/grade**	**D1853N ** **status**	**FH/consanguinity**
1	Peripheral	46	Female	Meningotheliomatous	-	+/+ 1^st^ cousin
	Central				+	
	Basal				-	
2	Peripheral	40	Male	Meningotheliomatous with micro- metastasis to the underlying tissue of brain	-	**-/-**
	Central				+	
	Basal				-	
3	Peripheral	21	Male	Meningotheliomatous/I	-	+/+ Pat 1^st^ cousin
	Central				-	
	Basal				-	
4	Peripheral	69	Female	Meningotheliomatous/I	-	**-/-**
	Central				-	
	Basal				-	
5		49	Male	Metastatic carcinoma (lung origin)	+	**-/-**
5a					+	

FH: Family history

The provided ages, as the complementary data, are related to the family members, as family history, within the pedigree of our patients Patient 1: Brother (brain tumor, onset: 40 years old) and Mother (brain tumor, onset/deceased: 70 years old). Patient 3: Three maternal uncles affected: 1^st^ with acute lymphoblastic leukemia at 65 years old; 2^nd^ with colorectal cancer; and 3^rd^ with leukemia.

The patients were referred from Department of Neurosurgery, Shariati hospital, Tehran, Iran, and the experimental work has been performed at Department of Medical Genetics, School of Medicine, Tehran University of Medical Sciences, Tehran, Iran.

Due to the personalized insight of this work, only the ratio analysis was, individually, provided. 

***Immunofluorescence (IF) assay:*** The extracted cells were washed with 1x phosphate buffer solution (PBS). Then, the cells were stained with primary antibodies including VEGF (Genature Europe, Belgium), and EGF (Novous Biologicals, USA). This mixture was incubated at 4 °C for 45 minutes, and washed with 1x PBS. The cells were stained using second-layer antibodies including phycoerythrin-indodicarbocyanine Cy5, and Rpe for VEGF and EGF, respectively. The status of PE was traced by the RXA2-fluorescence microscope (LEICA, DM, Germany). The average of 10000-20000 tumor cells was analyzed. The intensity of fluorescence was categorized as 1: low, 2: moderate, and 3: high, for both EGF (E) and VEGF (V) proteins.

## Results

**Figure 1:**
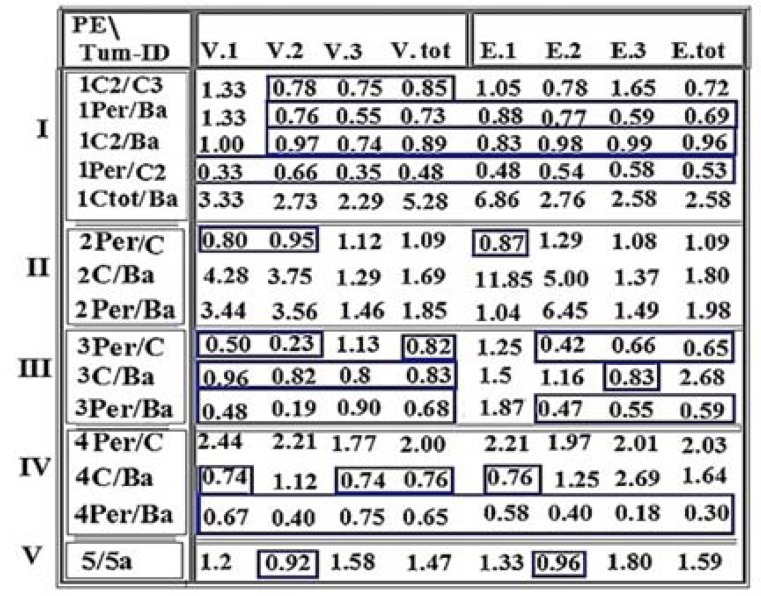
Ratio of protein expression of vascular endothelial growth factor (VEGF) and epidermal growth factor (EGF) between different sections of meningioma tumor and lung metastatic brain carcinoma (LMBC)

**Figure 2:**
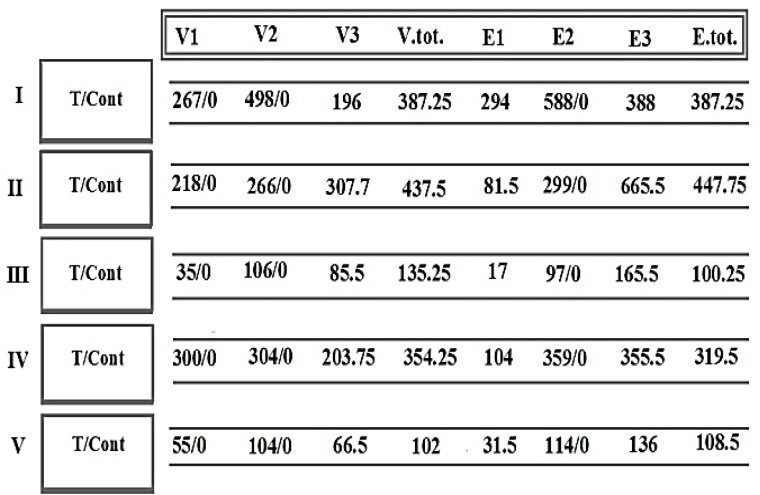
Ratio of protein expression of vascular endothelial growth factor (VEGF) and epidermal growth factor (EGF) between total tumor sections and the control

The mode of expression was categorized in the patients by focusing on the Per/C/Ba sections of tumors. Ratio distribution of PE for VEGF and EGF between different sections of four meningiomas and a LMBC tumor was provided (Figure 1). The comparative ratios with less than 1 value is displayed within the boxes, accompanied by the values more than 1. These indices reflect remarkable diversity between different tumor sections in all the patients (Figure 1). The Ratio of all tumor sections showed, diversely, higher index than in the control (Figure 2).

**Figure 3:**
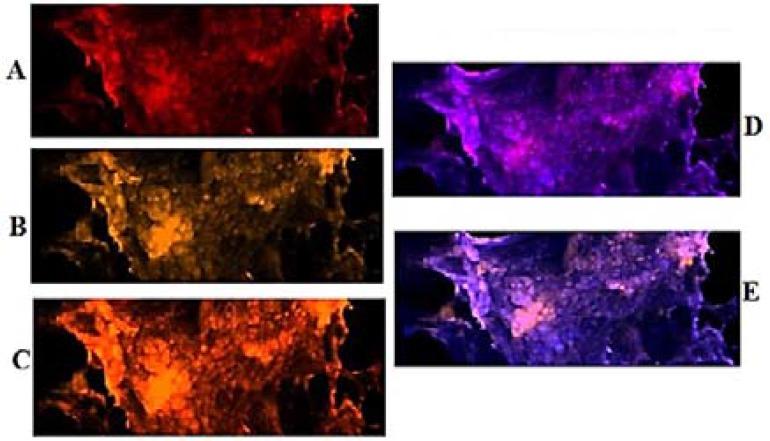
Protein expression of vascular endothelial growth factor (VEGF) and epidermal growth factor (EGF) in the meningioma cells from peripheral dissected region of tumor in patient 1. Respectively, A and B: cells conjugated with EGF and VEGF; C: co-expression of A and B; D and E: merged of dapi/EGF and dapi/VEGF

**Figure 4:**
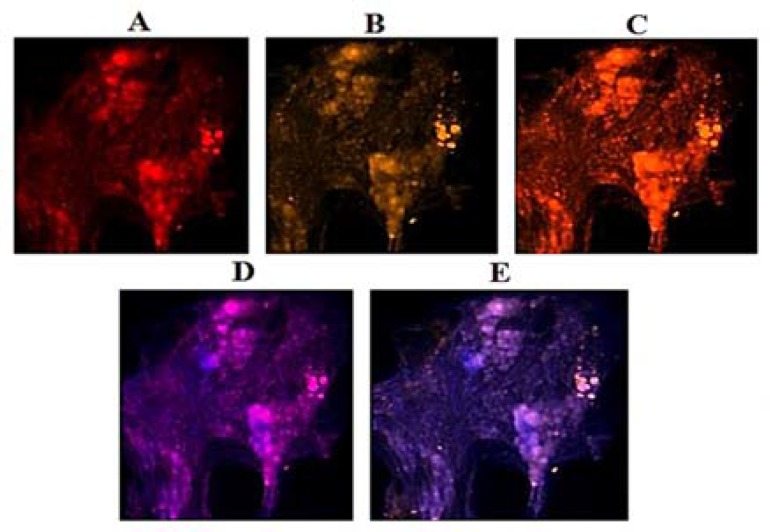
Protein expression of vascular endothelial growth factor (VEGF) and epidermal growth factor (EGF) in the meningioma cells from central-2 dissected region of tumor in patient 1. Respectively, A and B: cells conjugated with EGF and VEGF; C: co-expression of A and B; respectively, D and E: merged of dapi/EGF and dapi/VEGF

***Exploration of PE in VEGF and EGF: ***Patient 1: The per-section presented noticeable higher expression in VEG than EGF (Figure 3), and co-expression of two proteins was traceable (Figure 4).

**Figure 5:**
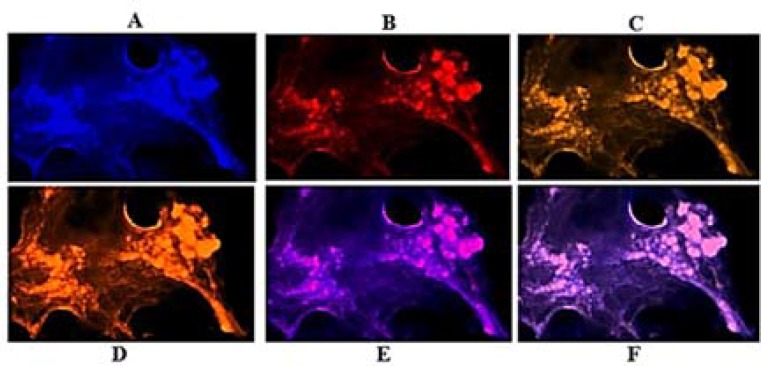
Protein expression of vascular endothelial growth factor (VEGF) and epidermal growth factor (EGF) in the meningioma cells from peripheral dissected region of fresh tumor in patient 2. A. meningioma cells with dapi; respectively, B and C: cells with EGF and VEGF; D: co-expression of B and C; respectively, E and F: merged of dapi/EGF and dapi/VEGF

**Figure 6:**
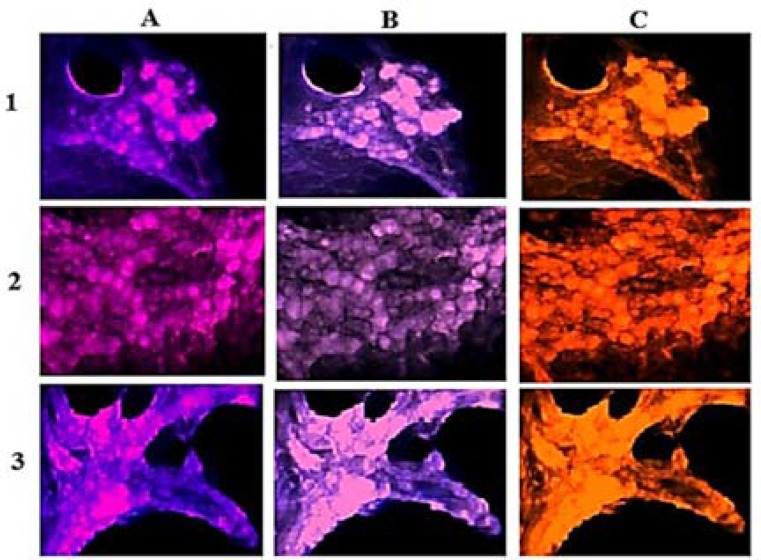
Protein expression of vascular endothelial growth factor (VEGF) and epidermal growth factor (EGF) in the meningioma cells from all sections of the fresh tumor in patient 2. Vertical text presents the original region of tumor samples, 1: peripheral; 2: central; 3: basal; horizontal text indicates the merged images, A: dapi/EGF; B: dapi/VEGF; and C: co-expression of EGF/VEGF

Patient 2: Tumor cells lacking PE in VEGF and EGF, and high PE was detected in more cells with VEGF in the per-section (Figure 5). The comparative PE between Per/C/Ba tumor regions was also diverse (Figure 6).

**Figure 7:**
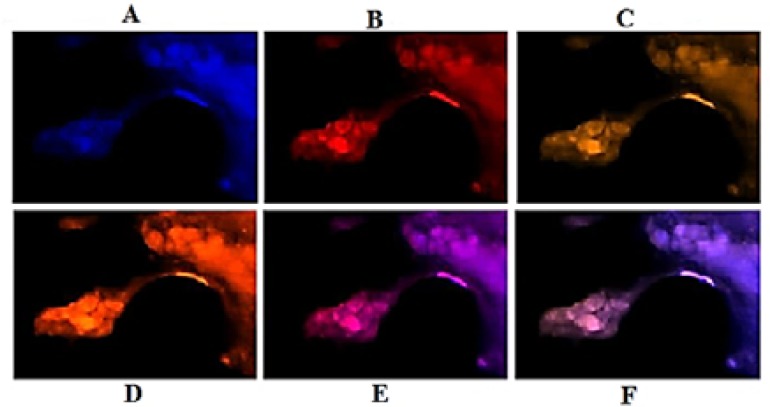
Protein expression of vascular endothelial growth factor (VEGF) and epidermal growth factor (EGF) in the meningioma cells from basal dissected region of fresh tumor in patient 3. Respectively, A, B, and C: Cells with dapi, EGF, and VEGF; D: co-expression of B and C; respectively, E and F: merged of dapi/EGF and dapi/VEGF

**Figure 8:**
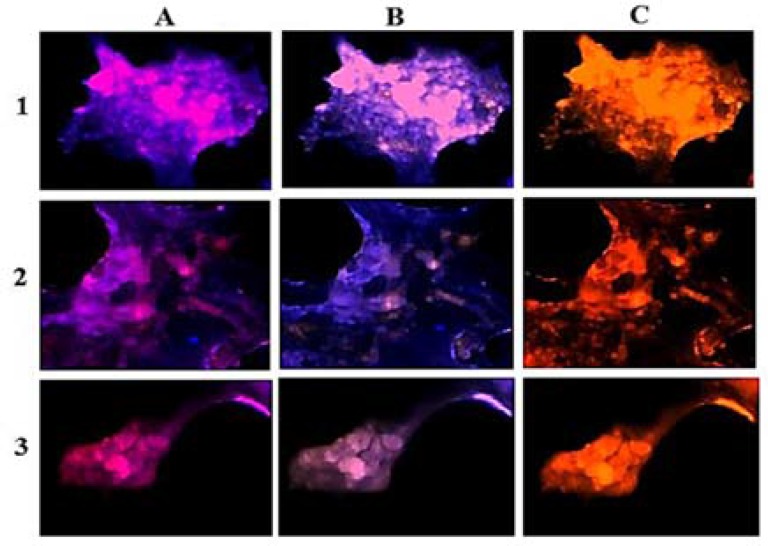
Protein expression of vascular endothelial growth factor (VEGF) and epidermal growth factor (EGF) in the meningioma cells from a unique section of the fresh tumor in patient 3. Vertical text indicates the region of tumor samples, 1: peripheral; 2: central; 3: basal; horizontal text presents the status of merged images, A: dapi/EGF; B: dapi/VEGF; and C: co-expression of EGF/VEGF

Patient 3: Lack of PE in C-section and co-expression of few cells were observed. The limited cells of Ba-section had high PE with harmonic co-expression (Figures 7 and 8).

PE: Protein expression; V.: VEGF; E.: EGF

I, II, III, IV, V are respectively related to the patients 1, 2, 3, 4, and 5.

Intensity of PE: V.1 and E.1: Low; V.2 and E.2: Moderate; V.3 and E.3: High; V.tot and E.tot: Total

C2/C3, C/Ba, Per/C, and C.tot/Ba are the ratios between different tumor sections in 5 patients.

**Figure 9:**
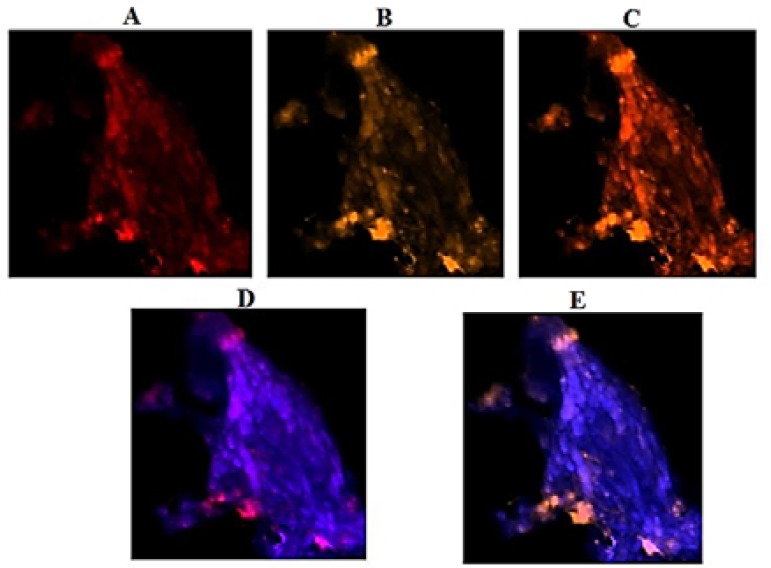
Protein expression of vascular endothelial growth factor (VEGF) and epidermal growth factor (EGF) in the meningioma cells from central dissected region of fresh tumor in patient 4. Respectively, A, B, and C: cells with EGF, VEGF, and merged of EGF/VEGF; respectively, D and E: merged of dapi/EGF and dapi/VEGF

**Figure 10:**
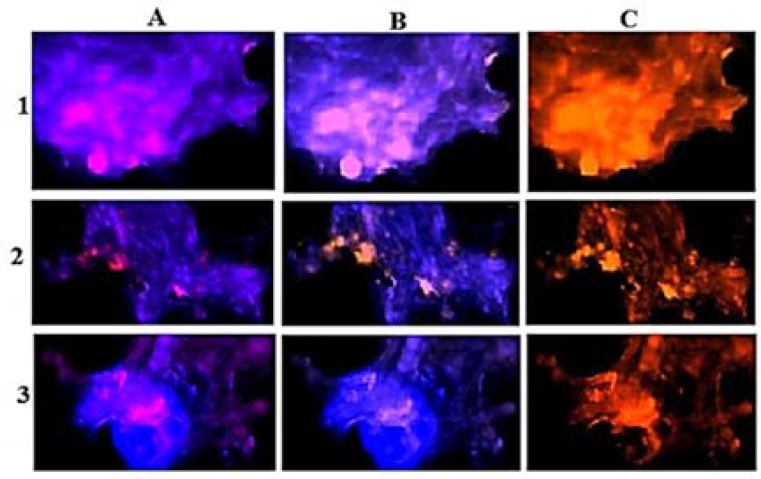
Protein expression of vascular endothelial growth factor (VEGF) and epidermal growth factor (EGF) in the meningioma cells from all sections of the fresh tumors in patient 4. Vertical texts indicate original region of tumor sample, 1: peripheral; 2: central; 3: basal; horizontal texts indicate status of PE images, A: dapi/EGF; B: dapi/VEGF; C: co-expression of EGF/VEGF

Patient 4: A minor clone of cells reflected high expression of VEGF, followed by lower expression of EGF in the C-section (Figure 9) with the diverse harmonic co-expression (Figure 10).

I, II, III, IV, V are respectively related to the patients 1, 2, 3, 4, and 5.

T/Cont: Ratio of tumor/control; V.: VEGF; E.: EGF

Intensity of protein expression (PE): V.1 and E.1: Low; V.2 and E.2: Moderate; V.3 and E.3: High; V.tot and E.tot: Total

**Figure 11:**
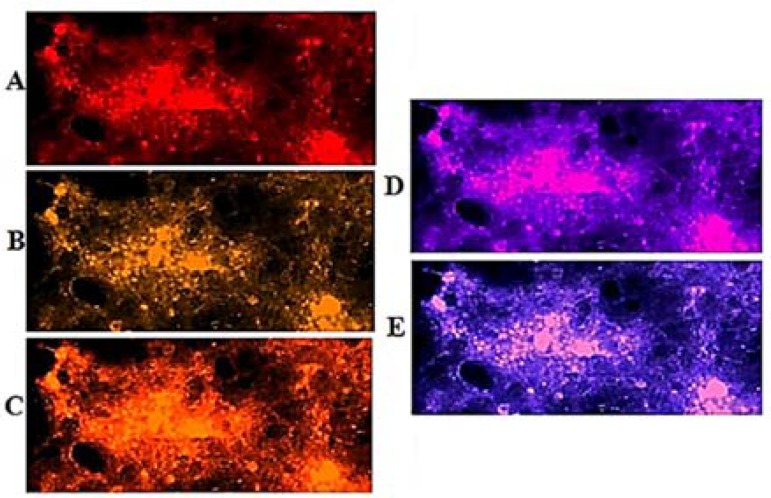
Protein expression of vascular endothelial growth factor (VEGF) and epidermal growth factor (EGF) in tumor cells of a patient with lung/brain metastatic carcinoma. Respectively, A and B: metastatic cells conjugated with EGF and VEGF; C: merge of EGF/VEGF; respectively, D and E: merge of dapi/EGF and dapi/VEGF

Patient 5: High expression was more frequent in EGF than in VEGF with a remarkable co-expression (Figure 11).

Scale bar: 20 µm

## Discussion

The qualitative and quantitative values may provide the prognostic value for characterization of each tumor section (Figure 1, A). 

Scale bar: 20 µm

Such strategy could be applied as the personalized- diagnostic, and prognostic insights for clinical managements including therapy. Therefore, tracing of any evolutionary pattern through the layers of each tumor is essential. 

In patient 1, degree of diversity was traced between two different C-subsections, for V2 and V3. It had, gradually, expanded to a higher level for the ratio of Per/Ba and C2/Ba. At the 3^rd^ step, evolution involved all intensity of these proteins in the ratio of Per/C2 (Figure 1, I). Interestingly, the molecular status of D1853N polymorphism, as a poor prognosis, was only positive in the C-section. 

Scale bar: 20 µm

In patient 2, diversity was observable for the expression ratio of Per/C in V1, V2 and E1 (Figure 1, II). Two elevated levels of the ratios for V1, E1, and E2 were remarkable for C/Ba ratio. An evolutionary course could be traced from peripheral to the center, and then to the basal sections (Figure 1). Similar to the patient 1, the positivity of D1853N in C-section, was also considered as an evolutionary event (Table 1).

Scale bar: 10 µm

Scale bar: 20 µm

In patient 3, the stepwise and harmonic altered mode of PE was predictable through lines 1 and 2 (Figure 1, III). Both VEGF and EGF were remarkably higher in C- than Per-section. Conclusively, the status of PE was higher in VEGF than in EGF for Per-section; but vice versa for C- and Ba-sections. 

Scale bar: 20 µm

Besides, by considering the predisposing role of D1853N and its status as negative, and the non-cooperative manner of expression for V and E proteins, a good prognosis might be predicted for this young patient. 

Scale bar: 20 µm

In patient 4, unique ratio was related to Per/Ba for all categories of intensity. In contrast, this value was partially similar to the ratio of C/Ba; and totally different to the ratio of Per/C which reflected stability of Ba-section (Figure 1, IV).

Scale bar: 10 µm

In patient 5, Ratio between two sections indicates the involvement of more frequent cells with PE in section 5 than 5a, which might be a sign of poor prognosis. By considering the tendency for up-regulating theory for cancer cells, such as the Per/Ba and C2/Ba ratios, the course of evolution seemed to be initiated from the basal section (Figure 1, I). 

Scale bar: 20 µm

In contrast, this process was initiated in central section for the ratio of C/Ba (Figure 1, II). However, the provided results varied from tumor to tumor, and was rather a personalized data. However, by considering the ratio of C-total/Basal, the ratio was higher in central- than basal-section, without any diversity. In LMBC, two unclassified sections were diverse for V2 and E2 than other indices. So, it might be stated that the cells with moderate intensity acted as GEAR or BRAKE for further progressive expression (Figure 1, V). Conclusively, there was a remarkable diversity between different 1) dual tumor sections in individual patient, 2) patients, and 3) ratios (Per/Ba, C/Ba, and Per/C) (Figures 1 and 2, III).

Variable diversity in the mode of PE play an important role in further developmental status of tumor. However, in patient 1, by comparing three different tumor sections, expression of EGF was lower than in VEGF for peripheral than central and basal sections of tumor (Figures 3 and 4). Co-expression of both proteins was a key channel through which the growth of tumor would be facilitated. So, an early preventive strategy could be considered.

In patient 2, cells with higher PE of VEGF than EGF in the Per-section, and a remarkable co-expression were informative (Figure 5, D). Besides, the cells with high expression of both proteins were related to basal section with a harmonic co-expression in all tumor regions as well (Figure 6). 

The challenging item in patient 3 was the harmonic cooperation of few cells with high PE in VEGF and EGF (Figures 7 and 8). The question was whether such limited cells with up-regulated function would be capable to facilitate the tumor progression or not. 

In patient 4, a minor clone of cells reflected high expression of VEGF, followed by lower expression of EGF in the central of tumor with notable co-expression (Figure 9-A, B, D, and E). Conclusively, higher expression pattern of VEGF than EGF may play an important role in the heterogenic nature of meningioma of tumor progression in peripheral section (Figure 10). So, the diverse PE for both proteins might have an impact on tumor behavior in this patient. The comparative images between peripheral, central, and basal tumor regions reflected the diverse expression in patient 4. In the Per-section, the high expression of both proteins, either as a sole or as the co-expressive manner, might direct the tumor behavior to an unexpected pattern.

In patient 5, the mode of expression of both proteins was also diverse. The cells had higher expression in EGF than VEGF, with a harmonic co-expression (Figure 11, A-E).

The complementary profiles of the key involved proteins in meningioma have been recently explored. It was shown that in spite of the low expression of Ki-67, and high expression of P14 and P63, there was a harmonic co-expression of Ki-67/P14/P63 in majority of cells.^15^ Moreover, another triangle protein profile included P53, ATM, and P63 which was indicative of a heterogenic expression pattern in these proteins, but with a harmonic interaction. By considering another profile, the highest expression in proliferating cell nuclear antigen (PCNA) and cyclin D2, and the lowest expression in cyclin E were observable in all types of brain tumors. However, high PE of proliferative factor played an important role in the tumor progression in brain tumors including meningioma.^15^

Besides, tracing the status of D1853N polymorphism is rather imperative, not just because of the positivity of this target, but due to the complementary molecular alteration(s) which may occur during the patients’ life. It’s worth to emphasize on the predisposing capability of this molecular alteration and the predictive impact on the post diagnostic management by being positive either for brain tumors or other neoplasms.^12^^-^^14^ A registered patent also indicates the involvement of D1853N alteration in all neoplastic disorders, with different incidences, and its’ predisposing impact on cancer managements (Mehdipour, Application number: PCT/IB2014/065072).^16^ Furthermore, the mosaicism of D1853N in patients 1 and 2 is a warning sign to predict an early management for the patients with cancer. 

Technical strategy and its application at cellular and/or molecular level plays a critical role in diagnosis of brain tumors. As an example, correlation between gene amplification at global level by qualitative polymerase chain reaction (qPCR) and expression by IF is reported to be a challenging item, and we found it inconsistent in brain tumors.^17^ This finding has a key impact on the therapeutic approach, so it’s worth to emphasize on an EGF/VEGF chimer characterized with an adequate amount of antibodies in contradiction of the EGF and VEGF function which aimed to prevent angiogenic and growth of breast tumor in mouse model.^18^

## Conclusion

The classified heterogeneity, diverse functional information, and harmonic co-expression in different territory of tumors may lead to predict the aggressiveness mode of tumors as a translational insight to the clinical managements including therapy in brain tumors. VEGF and EGF may be applied as the consistent biomarkers for brain neoplasms. Furthermore, applicability of anti-VEGF/EGF, as the multi-punitive strategy in therapy of brain tumor is required.

## Notes:

**How to cite this article:** Mehdipour P, Javan F, Faghih-Jouibari M, Khaleghi M. Diversity in tumor territory of meningioma: Protein expression in vascular endothelial growth factor and epidermal growth factor. Iran J Neurol 2019; 18(2): 43-9
